# Hytrel-like Copolymers Based on Furan Polyester: The Effect of Poly(Butylene Furanoate) Segment on Microstructure and Mechanical/Elastic Performance

**DOI:** 10.3390/molecules28072962

**Published:** 2023-03-26

**Authors:** Magdalena Kwiatkowska, Inez Kowalczyk, Zbigniew Rozwadowski, Elżbieta Piesowicz, Anna Szymczyk

**Affiliations:** 1 Department of Materials Technology, Faculty of Mechanical Engineering and Mechatronics, West Pomeranian University of Technology in Szczecin, Piastów Av. 19, 70-310 Szczecin, Poland; 2 Department of Inorganic and Analytical Chemistry, Faculty of Chemical Technology and Engineering, West Pomeranian University of Technology in Szczecin, Piastów Av. 42, 71-065 Szczecin, Poland; 3 Department of Technical Physics, Faculty of Mechanical Engineering and Mechatronics, West Pomeranian University of Technology in Szczecin, Piastów Av. 19, 70-310 Szczecin, Poland

**Keywords:** Hytrel-like copolymers, furan polyesters, thermoplastic polyester elastomer, mechanical properties, elastic recovery, bio-based polymers

## Abstract

This paper aims to compare the performance of two Hytrel-like segmented copolymers: “classic” PBT-b-PTMG and fully bio-based PBF-b-PTMG, containing poly(butylene furanoate) as the rigid segment. The idea behind this research is to assess whether the sustainable copolymers can successfully replace those “classic” once at the thermoplastic elastomers’ market. Two series of copolymers were synthesized under the same process parameters, had the same compositions, but differed in aromatic ring structure in terephthalate/furanoate unit. Furthermore, the materials were processed by injection moulding as typical Hytrel products. Then, the samples were subjected to extensive characterisation including NMR, GPC, FTIR, DSC, WAXS, DMTA, TGA techniques and mechanical tests with particular interest in the microstructure formed during processing and its effect on the copolymers’ mechanical and elastic behaviour. The detailed analysis proved that PBF-b-PTMG and PBT-b-PTMG copolymers represent two kinds of materials with similar chemical structure, some features of thermoplastic elastomers, but evident differences in their physical properties.

## 1. Introduction

Commercialization of thermoplastic elastomers (TPE) in the 1950s and 1960s became a major breakthrough in the plastic processing industry by extending a range of end-use product designs requiring specific rubber-like properties, whilst eliminating the inconveniences of the vulcanization process. Being able to combine the properties of rubber and engineering plastics with tunable performance, TPE still holds a high position in the plastics market with tendency to grow further [1,2].

A history of commercially available TPE starts with the development of segmented polyurethanes with relatively high tensile strength, good elastic recovery, high abrasion resistance, and processability typical for thermoplastics [3]. Further development resulted in the group of styrenic block copolymers (styrene and butadiene or isoprene) commercialized under Kraton trade name with stress–strain characteristics very similar to vulcanized styrene–butadiene rubber, but with higher tensile strength and resilience [3,4]. The third important group of TPE were thermoplastic polyester elastomers—the randomly segmented poly(ether/ester) block copolymers (TPEE), which have been produced by DuPont since 1972 as Hytrel^®^ (also Pelprene^®^ by Toyobo, Arnitel^®^ by DSM, Ecdel^TM^ by Eastman) [2,5]. The main advantages of these materials are high mechanical strength, good elasticity, significant dynamic properties, creep resistance, broad service temperature range, and excellent processability. All these features, which can be tailored by ester/ether segment’s ratio to meet the specified requirements to drive the high worldwide demand for TPEE.

Poly(ether/ester) copolymers are mainly composed of alternately arranged poly(butylene terephthalate) (PBT) rigid blocks and poly(tetramethylene glycol) (PTMG) flexible blocks connected by ester linkages. Such a combination of a crystallizable ester segment with a relatively high glass transition temperature, and an amorphous, highly flexible, ether segment with a low Tg that upon cooling from a melting temperature undergoes a micro- and nanophase separation, resulting in a multiphase structure [5,6,7,8,9,10,11]. In a solid state the crystal(line lamellar domains of PBT (the hard phase) form a more or less continuous network through the amorphous phase to provide strength and prevent its waning under stress. The soft phase is composed of ether segments mixed with some uncrystallized ester sequences and provides flexibility to copolymers. This specific microstructure is thermo-reversible, which ensures good processability of TPEE (injection, extrusion, compression, blow, rotational moulding, and highly precise products), whereas their exploitation properties depend on the content of each constituent. Due to a multi-function versatility of copolymers, poly(ether/ester) is suitable for many industrial applications, mainly in automotive and transportation, but also in medical devices and pharmaceutical packaging, food contact applications, machine design, electronics, furniture design, sporting goods, etc. [1,2,5,6,12,13]. Considering a very high consumption of TPEE materials and therefore, of the monomers for their synthesis, it is necessary to look for more sustainable counterparts based on biomass derived substrates. In addition, the great advantage of TPEs is that they are recyclable, and do not require additional chemical cross-linking reactions.

**Figure 1:**
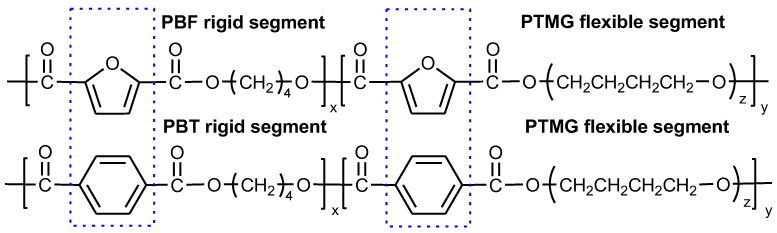
The chemical structures of two compared copolyesters: PBF-b-PTMG vs. PBT-b-PTMG.

Currently the furan polyesters, synthesized from plant-derived 2,5-furandicarboylic acid (FDCA), and specifically poly(butylene furanoate) (PBF), have a huge potential to replace PBT rigid segments [14,15,16], which when copolymerized with sustainable PTMG, lead to fully biobased TPEE [17,18]. The growing interest in research on furan polyesters results from the global trend in searching for monomers from renewable feedstocks, mainly green biomass [19,20,21]. This aims to protect the natural environment against overexploitation of fossil deposits, reduce energy consumption and greenhouse gas emissions during the production of both plant-derived monomers and polymers, as well as to achieve more efficient use of by-products in agricultural production. The investigations of the recycling of furan polyesters as well as their use in a closed loop are also very promising and beneficial for the natural environment [22,23]. Extensive research on furan polyesters reveals many similarities to terephthalate acid-based polyesters. However, in the case of thermoplastic elastomers, their performance depends on a state of phase separation driven by crystallization of the rigid segment. And in bio-based counterparts, their crystallizability is strongly affected by the specific structure of the furan ring and its non-linear connections with aliphatic chains, as well as higher polarity if compared to the benzene ring in the terephthalate unit [16,24,25]. Zhou et al. and Xie et al. described the performance of furan-ester copolymers based on PBF [26] or PEF [27] and PTMG and classified the materials as either tough thermoplastics or thermoplastic elastomers, depending on the rigid-to-flexible segment ratio. One of the aims of this paper is to compare the performance of two Hytrel-like copolymers: PBT-b-PTMG and fully bio-based PBF-b-PTMG synthesized under the same process conditions, having the same compositions, but differing only in aromatic ring in terephthalate/furanoate unit architecture (Figure 1). The novelty of this paper lies in the fact that in most studies on FDCA-based materials the measurements are performed on small quantities/pieces of the samples. In our research, the equipment used for the synthesis enabled us to produce the materials with an output of ca. 200 grammes, which was enough to process the copolymers by injection moulding as typical Hytrel products. Thus, the samples subjected to characterization can be considered as bulk (solid) materials with the microstructure formed during “standard” thermal processing as applied in industry. It provides a chance for a direct comparison of the performance of two kinds of copolymers with a particular focus on the effect of their microstructure on mechanical behaviour and elasticity. Thus, we aim to assess whether the sustainable copolymers can easily and successfully replace those “classic” ones in the area of thermoplastic elastomers application.

## 2. Results and Discussion

### 2.1. Chemical Structure of Copolymers

Two series of copolymers containing PBF or PBT as the rigid segment and bio-PTMG as the flexible segment were synthesized with different rigid-to-flexible segments ratios. In the copolymers’ abbreviations the number always stands for the rigid segment content. The materials were easily processed by injection molding and appear creamy-white in color with slight yellow tones for PBF copolymers. The calculated intrinsic viscosity values [η] for both series are comparable and range from 1.15 to 1.26 dL/g for PBF-b-PTMG and from 1.04 to 1.30 dL/g for PBT-b-PTMG with a tendency to grow with increasing PTMG content (Table 1). Considering the Mark—Houwink constants provided in [9] (K = 1.7 × 10^−4^, α = 0.83), the calculated average M_n_ values for PBT copolymers are between 36,490–47,740 g/mol. The number for average molecular weights (M_n_), determined by GPC techniques for the PBF-b-PTMG series, range from 36,900 to 69,100 with the PD index around 1.96–2.12. The resulting chemical structure and composition of materials were confirmed using Fourier-transform infrared spectroscopy (FTIR) and proton nuclear magnetic resonance (^1^H-NMR) techniques.

**Figure 2:**
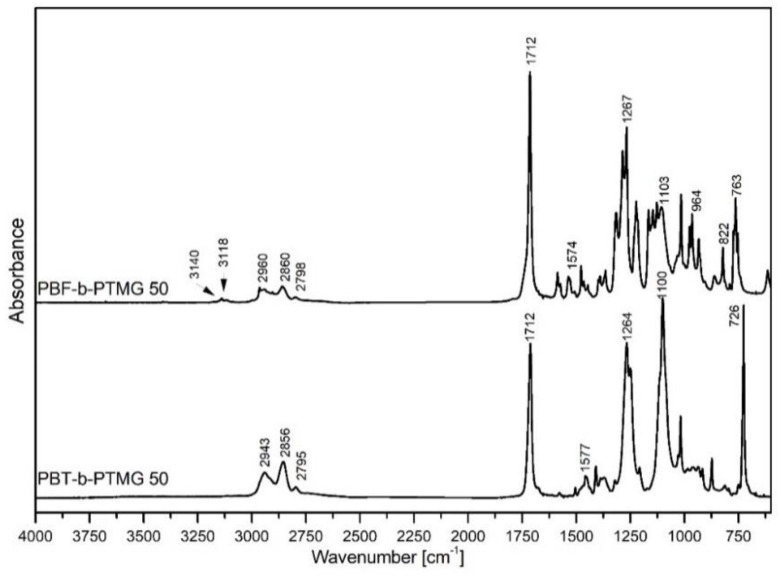
FTIR spectra of PBF and PBT copolymers.

The representative FTIR spectra for both kinds of copolymers containing 50 wt% of the rigid segment are presented in Figure 2, whilst all copolymers’ spectra are included in Figure S1. The absorption bands typical for polyester and polyether segments as well as the differences arising from different dimethyl esters used for copolymers synthesis and segments ratios can be observed [9,28,29,30]. Common absorption peaks can be observed at 1712 cm^−^^1^, which is attributed to C=O stretching vibration of the ester group and at about 1267 and 1100 cm^−^^1^, attributed to C-O and C-O-C stretching bonds. The absorption peak at 1574–1577 cm^−^^1^ comes from C=C stretching bonds in an aromatic ring. Additionally, some bands attributed to asymmetric and symmetric stretching vibrations in -CH_2_- groups from a butylene unit of PBF or PBT and PTMG segments can be detected at about 2854–2860 and 2940–2963 cm^−^^1^ wavenumbers. A small absorption peak detectable at about 2795–2798 cm^−^^1^ may be attributed to symmetric C-H streatching of the alkyl group [31,32]. For PBT-b-PTMG copolymers a strong band at ca. 726 cm^−^^1^ can be attributed to out-of-plane deformation in C–H of the aromatic ring [33], as well as alkane backbone [9,30]. In turn, the PBF-b-PTMG copolymers reveal the peaks typical for the furan ring: two weak signals at 3118 and 3140 cm^−^^1^, attributed to vibration mode of C-H bond, and the vibration modes at 964, 822, and 763 cm^−^^1^, associated with 2,5-disubstituted furans (it should be noted that the band at 763 cm^−^^1^ is bimodal, since C-H of the alkane backbond also contributes in this region). A lack of an absorption band at about 3400 cm^−^^1^, attributed to the terminal hydroxyl groups of PolyTHF^®^1000, confirms a successful polycondensation reaction between polyester and polyether segments, finally linked by the ester group [27].

**Figure 3:**
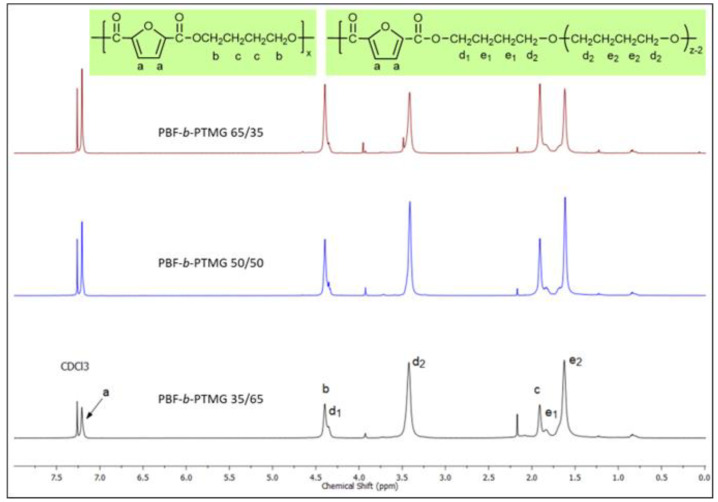
^1^H-NMR spectra of PBF-b-PTMG copolymers.

The ^1^H-NMR spectra of PBF-b-PTMG copolymers dissolved in CDCl_3_ are presented in Figure 3. Typical resonances for PBF segment can be distinguished as follows: at 7.21 ppm (peak a) corresponding to CH in the furan ring, at 4.40 ppm (peak b) and 1.91 ppm (peak c) corresponding to C(O)-OCH_2_- and -(CH_2_)_2_- methylene protons coming from the butylene unit. These resonance positions correspond to ^1^H-NMR spectra received for other PBF copolymers reported elsewhere [34,35]. As seen on spectra, the intensities of these peaks are increasing with the PBF segment content in copolymers. The resonances for the PTMG segment are detected at 4.35 ppm (peak d_1_) and 3.42 ppm (peak d_2_), and correspond to -OCH_2_- groups, whereby one is attached to the ester group of the furoate unit (d_1_), and second to the ether group of PTMG backbone (d_2_). Furthermore, two resonances at 1.83 ppm (peak e_1_) and 1.62 ppm (peak e_2_) correspond to aliphatic protons of inner -CH_2_- groups in the PTMG segment. These results are similar to the ^1^H-NMR spectra of PBF-PTMG copolymers reported by Zhou at al. [26]. The integral intensities of these two peaks (e_1_ and e_2_) were used to calculate real weight contents of the PTMG segment in copolymers, which are close to those calculated theoretically as reported in Table 1. The ^1^H-NMR spectra of PBT-b-PTMG copolymers included in SI (Figure S2, Table S1) also confirmed their chemical structure, and the real rigid/flexible segment contents are ca. 65.6/34.4, 49.6/50.4, and 38.1/61.9 wT%, respectively. Furthermore, the presence of the peaks d_1_, d_2_ and e_1_, which correspond to the PTMG segmental ends connected with PBF or PBT segments (in the case of PBT-b-PTMG copolymers -COO-CH_2_(d_1_)-CH_2_(e_1_)-CH_2_(e_1_)-CH_2_(d_2_)O-, Figure S2), constitutes evidence of a successful transesterification reaction between polyether -OH end groups and the ester groups in PBF (PBT) segments in a second step of polycondensation. Thus, the expected multiblocked structure of the PBF-b-PTMG and PBT-b-PTMG copolymers is confirmed.

### 2.2. Copolymers Microstructure and Thermal Behavior Analysis

**Figure 4:**
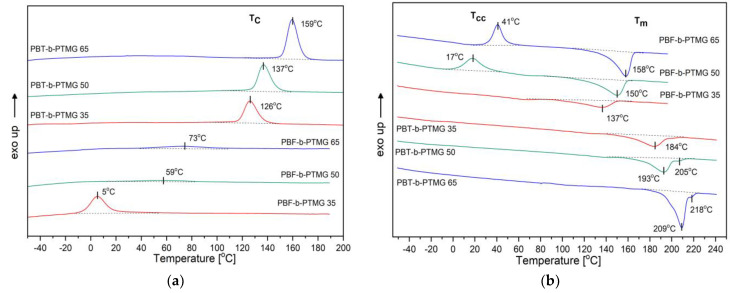
DSC thermograms of investigated copolymers: (**a**) cooling, (**b**) 2nd heating cycles, rate 10 °C/min.

When characterizing two kinds of multiblock copolymers, the first differences can be observed in their thermal behavior, and temperatures of phase transitions during the cooling and second heating cycles in differential scanning calorimetry (DSC) analysis (Figure 4 and Table 2). As expected, the PBT-b-PTMG copolymers, when cooled from melt with a standard rate (Figure 4a), crystallize in relatively high temperatures, i.e., 159–126 °C (T_c_), which decrease along with the PTMG content. Moreover, the crystallization process proceeds in a narrow temperature range. A lack of other exothermal peaks suggests that only the PBT segment is able to crystallize, and the lack of cold crystallization effects during subsequent heating (Figure 4b) proves that the crystallization rate of copolymers is fast enough to enable the materials to fully crystallize. In fact, the calculated crystallinity degrees (X_c_) range from 38 to 47%. The melting points (T_m_) are detected within 185 and 209 °C, and double melting peaks observed also strongly correspond to the nature of PBT, which is able to form two crystalline forms [5]. However, the bigger PTMG content the endothermal effects become broader. For PBT-b-PTMG 50 and 35 copolymers, the melting onset is observed at ca. 140 °C. It means that incorporation of flexible segments between PBT blocks does not impede the overall crystallization process in copolymers, but affects the quality of forming crystals, which are considered to be finer and more defected. All these observations align with the typical microstructure and thermal behavior of ether-ester block copolymers, in which phase separation is achieved by forming the PBT segment’s crystalline nanodomains of the hard phase dispersed in the PTMG-rich soft matrix (phase); a single glass transition is detected (T_α_ determined from DMTA analysis), and the transition temperatures are near those of polyester or polyether segments [5,7,8,9,34], but affected by the segment ratio.

The DSC traces for PBF-b-PTMG copolymers reveal significant differences in the crystallization process when compared to PBT materials with the same flexible segment contents. First, a considerable supercooling from the melt is required for the exothermal effects (T_c_) to appear within 5–73 °C range, though they are very weak (Figure 4a). Furthermore, the highest crystallization enthalpy, ∆H_c_, is observed for the copolymer with the smallest content of the rigid segment, which contrasts with PBT copolymers observations. When samples are subjected to a second heating cycle (Figure 4b), the PBF-b-PTMG 50 and 65 materials undergo cold crystallization with T_cc_ at 17 °C and 41 °C, respectively (thus the crystalline hard phase is further developed), and the melting temperatures are detected within 150–158 °C. In the case of PBF-b-PTMG 35, the T_cc_ does not appear, so the sample can be considered fully crystallized, and its melting point is observed at 137 °C. When explaining such behavior by the PBF copolymers, it should be noted that in multiphase materials the critical factor is which phase plays a role of the continuous matrix, thus dominating in the microstructure. In block copolymers, in general, flexible segments increase the mobility of the macromolecules, which facilitates their folding [5,6]. However, the DSC results prove that in furan–ester copolymers the rigid segments are so stiff due to their specific architecture, which, when dominating in the macromolecules, mutually restricts their mobility. It also restricts their diffusion, necessary in crystallization process.

As a consequence, the PBF segments crystallize in a hard form and incompletely, as observed in copolymers containing 65 wt% and 50 wt% of PBF, despite the presence of flexible segments. Different thermal behavior of PBF-b-PTMG 35 sample suggests a phase inversion in the copolymer microstructure, in which now the PTMG segment-rich soft phase makes the continuous matrix. And although a higher content of flexible segments leads to a decrease in the PBF average sequence length (DPx in Table 1). The whole macromolecules, in fact, gain in flexibility, which favors their diffusion and enables the rigid segments to interact. Although a very high supercooling is needed (∆T_m_ − T_c_ is ca. 130 °C) to initiate the crystallization process, due to a relatively low T_α_ (−44 °C, assigned to glass transition relaxation) the copolymer chains are mobile enough to form a crystalline phase, even at the temperature T_c_ of about 5 °C. Generally speaking, the phase separation is achieved in all investigated bio-based copolymers, thus PBF-b-PTMG materials reveal a heterogeneous structure typical for multiblock copolymers. However, their microstructure and performance are much more affected by a significantly lower crystallization kinetic being a consequence of the furanoate unit’s specific architecture. Furthermore, the temperatures of some physical transitions in PBF copolymers lay in application temperature range, which may also result in undesirable changes in their microstructure during exploitation.

Extending the thermal characterization of two series of copolymers, it is worth mentioning the thermo-oxidative stability of materials, applicable as thermally formed products. It is particularly important because, according to the literature, degradation of poly(ether/ester)s starts with an oxidation of relatively weak -C-O- bond in the ether segment, and further degradation proceeds according to the radical thermal oxidation mechanism [36]. By observing the thermogravimetric analysis (TGA) thermograms presented in Figure S3 and the degradation temperatures included in Table 2, one can see that both kinds of copolymers reveal two-step decomposition under air atmosphere: first, within 320–420 °C, and the second within the 425–500 °C temperature range, which is analogous to TGA results reported for PBF homopolymer and PBF-PEO copolymers [35]. Specifically, the 5% thermo-oxidative weight loss in PBF-b-PTMG samples is observed between 312 °C and 333 °C (T_dec 5%_), increasing along with the rigid segment content, whilst in PBT-b-PTMG it is slightly higher: between 344–350 °C. It is not surprising, since the thermal stability of PBT homopolymer is higher than PBF (378 °C vs. 350 °C) [34], but obtained results are also evidence that in both series the thermal stability is slightly deteriorated (referring to the homopolymer’s stability) by the presence of the ether segment. The most important conclusion from this analysis is that first decomposition reactions are observed far from the processing temperatures, which are usually 15–20 °C above the melting point. It means that all copolymers can be processed as typical thermoplastics, retaining their chemical structure and performance, and the effect of the rigid segment’s type is negligible in this case. However, it should be noted that the copolymers were stabilized with Irganox 1010, first to prevent PTMG thermal degradation during synthesis, and secondly to ensure relatively good thermo-oxidative stability of obtained copolymers.

Thermal analysis, although very useful in investigating copolymers’ microstructure, shows the nature of materials under controlled heating and cooling conditions. However, if the practical aspects of materials application are considered, the structure formed under the processing conditions and its effect on the copolymers’ performance are much more important. For that reason, the injection molded samples were subjected to wide angle X-ray spectroscopy (WAXS) two days and four weeks after processing, to assess their crystalline structure (also their stability), and through dynamic mechanical thermal analysis, (DMTA) to investigate their thermo-mechanical behaviour. As mentioned in the Section 3, polymers in melt were injected into a mold cavity having the temperature of 30 °C, which means that their cooling rate in the mold was higher than during DSC tests, and for PBF materials this temperature is also close to T_c_ and T_cc_ values.

**Figure 5:**
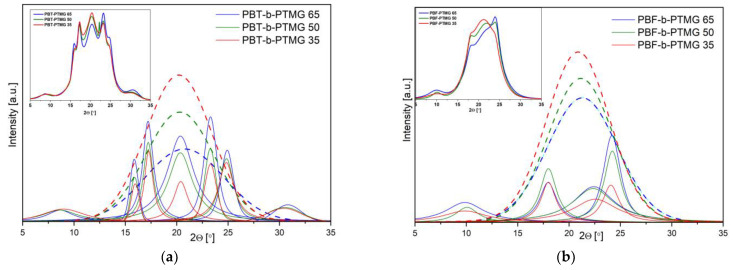
WAXS patterns of injection molded samples after deconvolution to crystalline peaks and amorphous halo for (**a**) PBT-b-PTMG and (**b**) PBF-b-PTMG copolymers (the original data presented in boxes). The spectra received after two days from processing. The amorphous halo peaks are in dash line.

The X-ray diffraction patterns after deconvolution show the prominent crystalline peaks of the crystallizable segments at the scattering angles (2*θ*) as follows: 8.8, 15.9, 17.3, 20.5, 23.4, and 25.1°, assigned to α-crystalline form of PBT (Figure 5a) [9,37], whilst the 10.1, 17.9, 22.4, and 24.2° positions are very close to those reported for triclinic α-form of PBF homopolymer, i.e., 10.66, 17.8, 22.4, and 25.03° (Figure 5b) [28]. The intensities of signals differ within the series of copolymers due to different content of the rigid segment; however, clear differences in the peak intensities, when comparing the PBT-b-PTMG and PBF-b-PTMG diffractograms, indicate variations in the crystalline structure development. Indeed, for PBF copolymers’ samples, tested two days after processing, the amorphous halos (marked in dash line) dominate the crystalline reflections, and the calculated values of mass crystallinity degrees (X_c_) vary from 26% to 40%, increasing with the rigid segment content (Table 2). In contrast the X_c_ for PBT copolymers range from 36% to 61%. These results confirm that due to slow and disturbed crystallization, the PBF copolymers are not fully able to crystallize during processing, which leads to unstable microstructure. As a consequence, the further (cold) crystallization process proceeds during the storage of molded samples in room or operating temperature, which may also result in undesirable shrinkage effects and changes of sample dimensions. This conclusion is supported by the WAXS spectra of samples stored in a bag at room temperature for four weeks (Figure S4). Slight but noticeable changes in peaks’ intensities are observed as well as an increase of calculated crystallinity degrees (29% to 48%).

**Figure 6:**
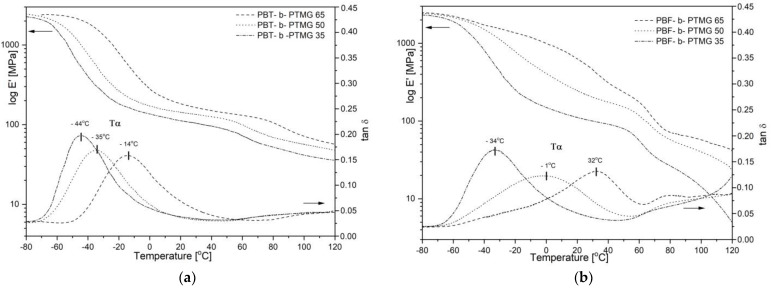
The storage modulus E’ and tan δ profiles for (**a**) PBT-b-PTMG and (**b**) PBF-b-PTMG copolymers.

Instability of microstructure in fully bio-based copolymers’ samples is also revealed in dynamic—mechanical tests (DMTA), particularly if compared to PBT copolymers’ behaviour. As seen in Figure 6a the storage moduli E’ of the PBT-b-PTMG samples reflect the typical regions resulted from viscoelastic nature, i.e., a glassy state at low-temperature range with values of about 2400 MPa, then a drop related to a glass transition of the amorphous phase, indicated by the maxima of the loss factor (α relaxation on tan δ), a broad rubbery plateau region within the temperature range of approx. −5 °C to 60 °C, and finally a gradual decrease related to approaching a melting temperature. Such E’ modulus profiles are observed for all copolymers, which confirms their features of thermoplastic elastomers, whereas a gradual decrease in E’ values and transition temperatures result from an increasing content of the PTMG segments. The presence of one distinct relaxation (α) on tan δ profiles from −44 °C to −14 °C is attributed to one glass transition of the flexible segment-rich soft phase; increasing heights of relaxation also indicate improved damping ability of copolymers with a larger PTMG content.

The storage modulus and loss factor profiles of PBF-b-PTMG copolymers are much more variable along with the temperature, and broader α relaxation peaks are observed (Figure 6b). In other words, the changes in copolymer chemical compositions result in much more pronounced differences in the material’s performance. Only the PBF-b-PTMG 35 copolymer (i.e., the one that seems to be fully crystallized from DSC) reveals thermo-mechanical behavior similar to Hytrel-like copolymers, with α relaxation max (glass transition) at −34 °C, and the rubbery plateau from ca. −20 °C to ca. 50 °C in temperature. This material is also characterized by the highest damping ability within the PBF series. In the case of copolymers with 50% and 65% of PBF content, a significant shift of α relaxation towards higher temperature is observed, and the values of T_α_ detected (ia. −1 °C and 32 °C) are far from the T_α_ values of PBT analogues. This observation seems to confirm the previously made conclusions about restricted mobility of macromolecules due to the furan unit stiffening effect. Copolymers need much more thermal energy to induce molecular mobility, and when passed through the glass transition region, polymer chains continue to form an equilibrium crystalline structure and stabilize. Since the differences between T_α_ and T_cc_ values are relatively small (−1 °C vs. 17 °C for PBF-b-PTMG 50, and 32 °C vs. 41 °C for PBF-b-PTMG 50), these two transition effects can superimpose, which makes the relaxation peaks on tan δ profiles broad and asymmetric. The temperature-induced changes in copolymers’ microstructure are reflected in the storage modulus E’ profiles, which do not reveal the rubbery plateau region, particularly in the sample with the highest PBF content. Furthermore, these transitions take place within assumed copolymers’ service temperature range, which is unfavorable, leading to changes in the copolymer performance.

**Figure 7:**
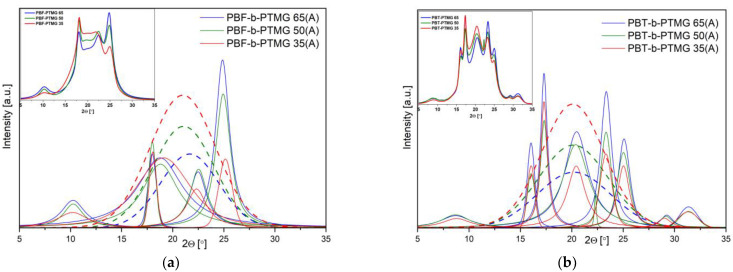
WAXS patterns of annealed injection molded samples after deconvolution to crystalline peaks and amorphous halo for (**a**) PBF-b-PTMG annealed at 70 °C and (**b**) PBT-b-PTMG annealed at 140 °C (the original data presented as inserts). The amorphous halo peaks marked in dash line.

For better insight into copolymers’ ability to crystallize, and thus to achieve phase separation, the injection-molded samples were subjected to thermal treatment, and the WAXS analysis of the investigated materials was performed once again (Figure 7). The PBF copolymers were annealed at 70 °C (i.e., less than T_m_, but higher than T_cc_ values) for 6 h under reduced pressure. For the comparison as well, the PBT materials were annealed at 140 °C. The changes in the crystalline structure development are visible at first sight (Figure 7a)—the original data show more variable profiles, and after deconvolution one new peak appears at ca. 18.8° of the scattering angle on all PBF copolymers’ spectra. The positions of the other peaks are practically the same as observed previously, whilst their heights and intensities have increased regarding the amorphous halo. As a consequence, the calculated values of mass crystallinity degrees for annealed samples vary from 48% to 71% along with the rigid segment content. It is interesting to note that the annealing also induced the development of the crystalline structure in PBT copolymers, which seemed to be fully crystallized (Figure 7b). The characteristic crystalline peaks kept the same scattering angle positions but became slender and more intense, and the crystallinity degrees increased to 44–72% (Table 2). Finally, the amount of the crystalline hard phase domains is comparable between two series of copolymers. Furthermore, the annealing process proves once again that processing conditions, particularly fast cooling from melt in the mold cavity, affect the copolymers’ microstructure, even those that are fast crystallizing.

### 2.3. Mechanical Behavior and Elasticity of Copolymers

**Figure 8:**
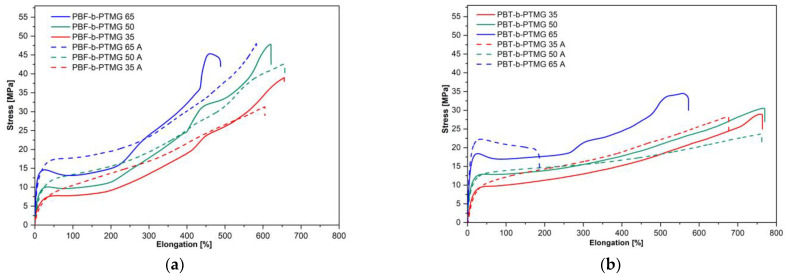
The representative uniaxial stress-strain plots of (**a**) PBF-b-PTMG and (**b**) PBT-b-PTMG copolymers after one month of storage and after annealing (A).

As mentioned above, the purpose of this paper is to compare the performance of two kinds of Hytrel-like copolymers or, in other words, to assess whether a replacement of PBT segments by bio-based PBF blocks would result in materials with mechanical and elastic properties similar to those of thermoplastic elastomers. Previous analysis of copolymers’ physical transitions demonstrated a significant influence of the furanoate units as well as microstructure instability. Thus, some differences in mechanical behaviour were also foreseen. The mechanical properties of investigated materials were analyzed within static and cyclic tensile tests and Shore D hardness. In order to get a broader view of changes in copolymers’ deformability (due to microstructure development), particularly PBF-b-PTMG, the tensile mesurmenets were performed for samples at different times from injection molding: after 48 h, one week, four weeks, and after the annealing process. The stress-strain characteristics are depicted in Figure 8 and Figure S5, whilst the mechanical parameters were collected in Table 3 and Table S2.

When two series of copolymers (after one month of storage) are evaluated, one can see that in tensile tests the samples reveal various levels of stress, particularly at yield, dependent on rigid-to-flexible segment ratio as well as high ability to deformation (Figure 8, Table 3). Although the σ_y_ values are quite similar for PBF and PBT analogues (7.4 MPa to 14.4 MPa vs. 9.5 MPa to 18.4 MPa), the same applies to Shore hardness (40.0 ShD to 52.2 ShD vs. 41.7 ShD to 50.4 ShD), significant differences are observed for maximal tensile stress (R_m_) values, recorded as stress at break. This is due to the different behaviour of materials under tensile force. Substantially, for all PBF copolymers the stress-strain curves have a shape typical for amorphous or low-crystalline polymers: with clear yield at elongation of ca. 21–40%, then necking, and finally, the gradual but conspicuous increase of the stress and strain level up to a sample’s break. Such behaviour indicates an orientation and alignment of macromolecules along with a tensile load, or in other words, stress induced crystallization, which results in a 3–4 fold growth of stress values if compared to σ_y_. Copolymers also reveal relatively high elongations within 477–657% with increasing PTMG content. When analysing the stress-strain paths of PBT copolymers, only the PBT-b-PTMG 65 curve refers to PBF materials’ behaviour but with a lower strengthening effect. The others correspond more closely to elastomer curves, i.e., samples that extend easily with slight increases of stress, no necking effects, a lower ultimate tensile stress level (R_m_ 25.5–34.5 MPa vs. 38.1–45.4 MPa), but with elongations at break of ca.100% larger than for PBF analogues. In general, the PBT samples deform uniformly, which seems to be related to the quality of microstructure formed. As mentioned above, the specific mechanical behaviour of thermoplastic elastomers results from the heterogeneous microstructure, in which crystalline nanodomaines of the hard phase are embedded in a deformable soft phase. According to the WAXS analysis, the crystallinity degrees of PBF-b-PTMG copolymers are relatively low (even after storage, X_c_ values are lower than for PBT copolymers), which means that in their microstructure the amorphous soft phase doped with rigid segments dominates (insufficient phase separation). Therefore, under tensile stress, macromolecules have more ability to disentangle and stretch out, whilst in PBT copolymers crystals act as physical nodes resulting in more elastomeric behaviour. It should be pointed out, however, that when comparing the tensile curves of the samples at different time intervals, neither PBF nor PBT copolymers show significant differences in the shapes of curves or in mechanical parameters’ values (Figure S4, Table S2). The instability of the microstructure concluding from thermal and WAXS analysis in the previous Section (also the detectable cold crystallization) does not significantly affect the mechanical performance of copolymers with different compositions. The one exception is the PBF-b-PTMG 35 sample tested in 48 h from injection molding, whose stress-strain path has a different shape, showing higher stiffness (E modulus) and reduced ability to deform. This material contains the smallest PBF segment content; furthermore, its crystallization was detected below room temperature (the same temperature as the injection mold), thus it can be presumed that its microstructure could have evolved more than in the other copolymers. The effect, however, of the annealing process on the mechanical performance of investigated materials, especially PBF copolymers, is noteworthy. As Figure 8a shows, the character of the tensile curves for annealed samples becomes more elastomeric, especially in the initial range—for copolymers containing 35 and 50% of the PBF segment, the yield and necking are no longer observed, and the E modulus values are reduced. The copolymers do not undergo such a strengthening effect, but the elongation at break’ values are comparable. These changes can be explained by the development of the crystalline structure and the forming of secondary crystals—thus, the phase separation, resulting from thermal treatment and cold crystallization. This was previously confirmed by the WAXS analysis. It can also be expected that the crystalline nanodomais are finer, which should be profitable for the elasticity of the copolymers. In the case of the PBT-b-PTMG materials, the differences, although detectable, are not significant, though the stress-strain curves also gain a more elastomeric character. This results from the development of the crystalline structure as well, confirmed by an increase in crystallinity degree after annealing (Table 3). Only for the copolymer with the highest PBT content are changes in mechanical behaviour observed. Although the E modulus’ values are comparable, a significant decrease in elongation and an increase in σ_y_ are revealed. Similar behaviour in PBT and PBF copolymers modified with 35% and 20% of dimerized fatty acids were reported [34]. In this case it was explained that a growth of crystalline nanodomains constrained the continuous soft phase and reduced the ability of material to become deformed.

**Figure 9:**
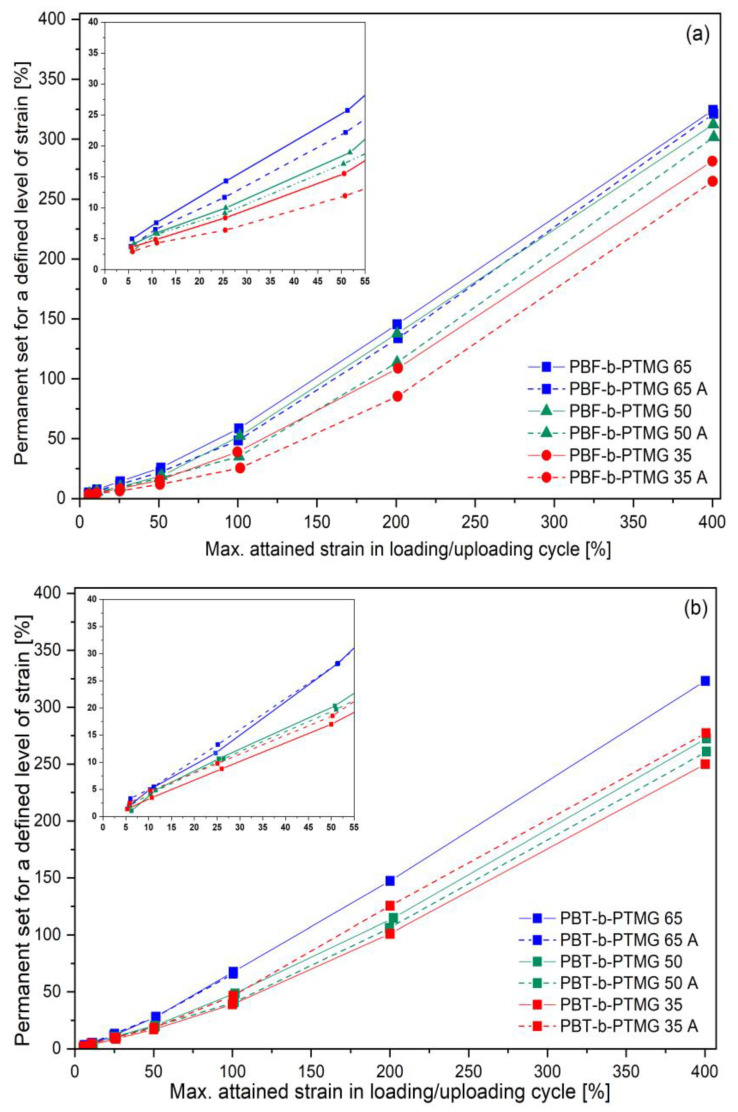
Permanent set values after loading/unloading cycle up to 5, 10, 25, 100, and 200% of the attained strain for PBF (**a**) and PBT (**b**) copolymers. Samples after one month of storage. Dash lines refer to annealed samples.

The cyclic tensile tests of copolymers provide knowledge on their elastic deformability and reversibility by an assessment of permanent set values remaining after every loading/unloading cycle within a defined strain range. Details about tests performed were described elsewhere [35,38], whereas the cyclic stress–strain curves, as well as the values of the permanent set at different strain levels for both series of copolymers, also annealed, are shown in Figure 9 and Figure S6. When analysing the responses of two series to cyclic deformation it can be seen that every following cycle is reflected by a hysteresis-like loop, whose shape strongly depends on achieved stress and residual strain levels (Figure S6a,b). The outlines of cyclic paths for investigated materials follow the profiles of their corresponding curves, received under static tensile experiments. Similarly, an increase in stress in subsequent cycles is slightly higher for PBF copolymers and for the annealed samples. The ability for elastic recovery of two series of materials reveals relatively small but noticeable differences, mostly for PBF copolymers, which seems to be affected by the resultant microstructure. For PBT copolymers at the initial stage of deformation (up to ca. 10–15% of max. attained strain), the reversibility is relatively high (with the permanent set values within 3% to 5%), and practically the same for all samples, regardless of PTMG content or thermal process (Figure 9b). With increasing strain level, the growing differences in deformability between the PBT-b-PTMG 65 copolymer and other samples are observed, particularly when 25% of strain is exceeded, which describes the material with the smallest flexible segment content, the amorphous soft phase. In addition, the yield point on the static stress-strain path is observed for ca. 27% of elongation. The other PBT copolymers reveal very comparable values of residual strain of ca. 9–12% for ca. 25% of attained strain, and ca. 17–20% for ca. 50%. Then the differences among copolymers become more pronounced, although the samples are also distinctly deformed. However, there is no clear influence of the annealing on materials’ elasticity—for PBT-b-PTMG 50, it is slightly improved, and for PBT-b-PTMG 35 the residual strain is slightly higher. In turn, more explicit effects are observed for PBF copolymers (Figure 9a): Primarily, the elasticity of materials is more affected by the increasing PTMG content (more flexible segments lower residual strain); in addition, the annealing process reduces the permanent set values at different attained strain levels, thus improving the copolymers’ capability of elastic recovery. These effects are not significant but detectable. Although the static stress–strain characteristics of the copolymers indicate their semicrystalline rather than elastomeric character, the permanent set values at defined strain level are comparable to those of PBT materials: ca. 5–8% for 10% of attained strain, ca. 8–10% for 25% of strain, and ca. 15.5–19% for 50%. It is evident that the elasticity of two series of copolymers is practically at the same level, although the structural investigations above indicated significant differences in the copolymers’ microstructure due to low crystallisation ability and instability of PBF segments. Furthermore, the decrease in residual strain for annealed samples is in accordance with the annealing effects observed in static tensile tests: changes in the shape of deformation paths to a more elastomeric quality were explained by crystalline structure development. At the same time, it can be assumed that crystalline nanodomains of the hard phase dispersed in the continuous soft phase, are finer, thus do not confine its elastic deformability, but support reversibility as physical crosslinks. These effects are opposite to those reported for annealed PEF/dimerized fatty acid diol copolymers [38], in which a development of phase separation resulted in reducing elastic deformability of materials. However, the reason could lie in different features of the soft segments, namely length (PTMG 1000 g/mol vs. FADD 570 g/mol) and intrinsic conformational flexibility.

## 3. Materials and Methods

### 3.1. Materials

The monomers for the copolymer synthesis were as follows: 2,5-furandicarboxylic acid dimethyl ester (DMFDCA), purity 99% (Matrix Fine Chemicals GmbH, Flums, Switzerland), terephthalic acid dimethyl ester (DMT), purity 99% (Sigma Aldrich, Steinheim, Germany), renewable 1,4-butanediol (bio-BD) and poly(tetramethylene glycol) (bio-PTMG), PolyTHF^®^1000 with the molecular weight of 1000 g/mol (both kindly supplied by BASF SE, Ludwigshafen, Germany). Tetrabuthyl orthotitanate (Ti(OBu)_4_) (Fluka) was used as a catalyst, and Irganox 1010 (BASF SE, Ludwigshafen, Germany) as antioxidant. The solvent: phenol/1,1,2,2-tetrachloroethane mixture (60/40 by weight) was purchased in Sigma Aldrich, Steinheim, Germany.

### 3.2. Synthesis of PBF and PBT Based Copolymers

The investigated copolymers have been prepared in a similar manner, as previously studied: PBF-b-FAD [34], and PBF-b-PEO [35]. For the synthesis, a two-step polycondensation in melt was applied. Two series of materials were synthesized under the same procedure, but with different temperature ranges, using a 1 dm^3^ capacious steel chemical reactor. Because the molecular mass of PTMG segment was constant, the changes in the copolymers’ segment ratio were modified by PBF or PBT segment length. In the first step a transesterification reaction between specified dimethyl ester and bio-BD (1:2 molar ratio respectively), catalyzed by Ti(OBu)_4_ (0.25% in relation to the ester in total), was produced first, and methanol was released as the by-product. The temperature range was 160–180 °C for the PBF segment, and 160–190 °C for PBT. In the second step, an appropriate amount of bio-PTMG (regarding rigid to flexible segment ratio), with additional part of the catalyst and antioxidant (0.5 wt% in relation to the copolymer final mass), were introduced into the reactor, and a melt polycondensation was carried out under a reduced pressure (25–30 Pa) and 1,4-BD release. For this step, the temperature range was 190–225 °C for PBF-b-PTMG, and 210–250 °C for PBT-b-PTMG copolyesters. The process was carried out until reaching an increase in specified value of stirrer torque due to an increase of the reaction mixture viscosity. Finally, the polymer melt was extruded from the reactor into a water bath, dried, and granulated. The material output was ca. 150–160 g.

In this study the PBF-b-PTMG and PBT-b-PTMG copolymers with 65, 50 and 35 wt% of the rigid segment are characterized. The experiments were performed on injection molded dumbbell-shaped samples (ISO 37:2005, type 3, 60 mm in length, a rectangular cross section 2 *×* 4 mm^2^) prepared using the injection molding machine Boy 150 (Dr. Boy, Neustadt-Fernthal, Neustadt, Germany). The molding temperature was 15–20 °C higher than the melting point on DSC thermograms, and the pressure 40–60 MPa was applied. The mold temperature was 30 °C.

### 3.3. Characterization Methods

The intrinsic viscosity measurements [η] were performed on capillary Ubbelohde viscometer (type Ic, K = 0.03294) for 0.5 g/dL concentrated polymer solutions in phenol/1,1,2,2- tetrachloroethane (60/40 by weight). The viscosities were measured at 25 °C. The molecular weights of PBF-b-PTMG materials were determined by GPC in hexafluoroisopropanol (HFIP) on a system equipped with Waters 1515 Isocratic HPLC pump, Waters 2414 refreactive index detector (35 °C), Waters 2707 autosampler, and PSS PFG guard column, followed by two PFG-linear-XL (7 μm, 8 *×* 300 mm) columns in a series. The columns were kept at 40 °C with flow rate 0.8 mL/min. The molecular weights were calculated against PMMA standards.

The chemical composition of copolyesters was confirmed using both Fourier-transform infrared spectroscopy (FTIR) and proton nuclear magnetic resonance (^1^H-NMR) techniques. The attenuated total reflectance ATR- FTIR spectrophotometer Tensor-27 (Brucker, Ettlingen, Germany) equipped with a germanium crystal ATR accessory was applied. The wave number range of 4000–600 cm^−^^1^ was used to get spectra, which were further normalized to the absorption band at 1712 cm^−^^1^. For ^1^H-NMR analysis Bruker 400 MHz spectrometer was used. The PBF-b-PTMG samples were dissolved in CDCl_3_ after extraction in methanol. The real fractions of the PTMG segments in copolymers were calculated based on the characteristic peaks’ integral intensities, according to Equation (1) found in [26]:

(1) $$W_{PTMG}\left(wt\%\right)=\frac{\left(\frac{I_{e1}}{4}x\;88\right)+\left(7\frac{I_{e2}}{4}\;x\;72\right)}{\left(\frac{I_{a\;}}{2}\;x\;210\right)+\left(\frac{I_{e2}\;}{4}\;x\;72\right)}100\%$$

where: *I_a_*_,_ *I_e1_*, and *I_e2_* are the integral intensities of a, e_1_ and e_2_ resonances on ^1^H-NMR spectra, whilst 210, 72, and 88 are the molecular weights of repeating units in PBF and PTMG, and O(CH_2_)_4_O sequence in the PTMG segment, respectively.

The thermal transitions in polymer materials were studied with differential scanning calorimetry (DSC, NETZSCH DSC 214 Polyma, Germany) applying heating–cooling–heating cycles within the temperature range −60–50 °C, and standard rate of 10 °C/min. The melting and crystallization/cold crystallization temperatures were determined as maximums of endo-/exothermal peaks. The crystallinity degrees (x_c_) of copolymers were calculated according to Equation (2):

(2) $$X_{c}=\frac{\Delta H_{m}-\Delta H_{Cc}}{w\times \Delta H_{m}^{o}}\;100\%$$

where: Δ*H_m_*—melting enthalpy, Δ*H_Cc_*—cold crystallization enthalpy (if observed), Δ*H^o^_m_*—heat of fusion of fully crystalline polyester segment, *w*—weight fraction of the rigid segment. For PBF Δ*H^o^_m_* = 129 J/g [15] and for PBT Δ*H^o^_m_* = 142 J/g [39]. The thermal stability, in turn, was measured on thermogravimetric balance (TGA 92-16.18 SETARAM Instrumentation, France), with the heating rate of 10 °C/min and temperature range 25 to 550 °C under air atmosphere.

In order to evaluate a dynamic–mechanical behavior and relaxations in two series of copolyesters the dynamic mechanical thermal analysis (DMTA) was performed using DMA Q800 thermal analyzer (TA Instruments, New Castle, DE, USA). The samples were examined in multi-frequency strain module with a frequency of 1 Hz and the temperature range −90 to 120 °C. The heating rate was 3 °C/min. The maximum temperature of α-relaxations (T_α_) attributed to glass transitions in copolymers were determined from tan σ profiles.

The wide-angle X-ray diffraction analysis (WAXS, X’PERT PANALYTICAL) was used to examine changes in crystalline structure of copolymers’ samples after different time shifts and treatments. Measurements were performed using CuK_α_ radiation lamp (0.154 nm) in the range of 0—40° scattering angle. The spectra were subjected to analysis by WAXSFIT software [40] for a deconvolution of the crystalline peaks and materials crystallinity degrees (X_WAXS_) calculation.

The mechanical properties: tensile strength and elastic deformability/reversibility of copolyesters were examined using the universal testing machine (Autograph AG-Xplus, Shimadzu, load cell 1 kN) with TRViewX video extensometer on injection molded samples as mentioned above. The mechanical parameters, i.e., tensile strength (R_m_), elongation at break (ε_b_), tensile modulus (E), and stress at yield (σ_y_) if observed, were determined in uniaxial tensile tests according to ISO 527-1,2: 2012 with the deformation speed of 100 mm/min. The minimum number of tests done was six. The elastomeric behaviour of materials, in turn, was evaluated applying cyclic loading and unloading procedure clearly described in [35,38]. A polymer sample was subjected to extension up to a defined level of strain (loading) and fully released to zero tensile force (unloading). The cycles were repeated successively with gradual increases in strain level (5, 10, 25, 50, 100, 200 and 400%). The permanent set values were determined as an increment of sample length after following loading/unloading cycle.

The hardness of copolymers was evaluated using the Shore test on durometer type D (Zwick) after 15 s of loading. At least ten measurements were taken. The density of injection-moulded samples was determined on the hydrostatic balance (Radwag WPE 600C, Radom, Poland) at 23 °C.

## 4. Conclusions

In conclusion, the extended investigations of fully bio-based Hytrel-like copolymers revealed clear differences in the performances of the two series of copolyesters, which undoubtedly stem from variations in terephthalate/furanoate unit specific architectures. The PBF-b-PTMG materials reveal lower melting temperatures and hindered crystallization, which will affect their processing conditions, and maximum operating temperatures. Any further consequence of a difficult crystallization is insufficiently developed, and unstable crystalline structure, thus phase separation, formed in thermally processed samples (parts). This specific heterogeneous microstructure may evolve on its own during storage due to cold crystallization effects, as proved by WAXS analysis. The most evident differences between PBT and PBF copolymers are observed in their mechanical performance, mostly under uniaxial tensile force. The PBF-b-PTMG characteristics have the shape typical for amorphous or low-crystalline polymers rather than elastomers (like PBT-b-PTMG), with significant strengthening effects. This is explained by the low content of the crystalline hard phase domains, which in thermoplastic elastomers act as physical nodes preventing macromolecules to flow. This behaviour, however, can be modified by thermal annealing process, which was proved to enhance copolymers crystallinity and phase separation, resulting in more elastomeric character. Despite the clear differences in static tensile behaviour, surprisingly, the elastic deformability of two kinds of copolymers turned out to be comparable, with similar permanent set values at specified strain levels for PBF and PBT copolymer analogues, slightly reduced by annealing. Finally, the investigations reported in this paper prove that PBF-b-PTMG and PBT-b-PTMG copolymers represent two kinds of materials with similar chemical structure, some features of thermoplastic elastomers, but evident differences in their physical properties. The specific architecture of the furan ring and furan ester segments affect the overall macromolecular structure of the copolymers, which means that fully bio-based PBF segments cannot directly replace “classic” PBT blocks in commercial thermoplastic copolymers. However, more environmentally friendly PBF-b-PTMG copolymers due to their performance can also find a place at the plastic market, due to their performance.

## Figures and Tables

**Table 1:** The chemical composition and molecular weights of PBF-*b*-PTMG copolymers.

Sample	In Feed			^1^H-NMR					[η]	GPC	
	DPx	W_PBF_	W_PTMG_	Integral Intensities			W_PTMG_	W_PBF_		M_n_	PDI
	Mol	wt%	wt%	*I_a_*	*I_e1_*	*I_e2_*	wt%	wt%	dL/g	g/mol	
PBF-b-PTMG 65	9.90	65	35	1.00	0.48	2.35	35.88	64.12	1.15	36,900	2.01
PBF-b-PTMG 50	5.33	50	50	1.00	0.60	4.13	48.88	51.19	1.19	60,970	2.12
PBF-b-PTMG 35	2.87	35	65	1.00	0.68	7.72	63.10	36.90	1.26	69,100	1.96

DPx—degree of polymerization of PBF segment, W_PTMG_—weight content of flexible segment, W_PBF_—weight content of rigid segment, [η]—intrinsic viscosity, M_n_—the number average molecular weight, PDI—polydispersity index.

**Table 2:** Thermal parameters and degrees of crystallization determined for PBF and PBT copolymers.

Sample	T_α_ [°C]	T_Cc_ [°C]	ΔH_Cc_ [J/g]	T_m_ [°C]	ΔH_m_ [J/g]	Tc [°C]	ΔH_C_ [J/g]	x_c_^DSC^ [%]	x_c_^WAXS^ [%]	T^Air^_dec_	
										T_dec 5%_ [°C]	T_dec max_ [°C]
**PBF-b-PTMG 65**	32	41	17	158	32.7	73	6.23	19	40 (71)	333	385
**PBF-b-PTMG 50**	−1	17	18	150	24.7	59	2.4	10	36 (59)	328	384
**PBF-b-PTMG 35**	−34	-	-	137	13.9	5	22.1	31	26 (48)	312	380
**PBT-b-PTMG 65**	−14	-	-	209/218	35.1	159	36.8	38	61(72)	350	397
**PBT-b-PTMG 50**	−35	-	-	193/205	33.0	137	29.2	47	50 (60)	345	396
**PBT-b-PTMG 35**	−44	-	-	185	22.2	126	26.0	45	36 (44)	344	398

T_α_—temperature of α-relaxation peak at tan δ (DMTA); T_cc_- cold crystallization temperature; ΔH_cc_—enthalpy of cold crystallization; T_m_—melting temperatures; ΔH_m_- enthalpy of polymer melting; T_c_—crystallization temperatures; ΔH_c_—enthalpy of crystallization, x_c_^DSC^—crystallinity degree of hard phase calculated from melting enthalpies; x_c_^WAXS^—crystallinity degrees calculated from WAXS (after annealing in brackets); T_dec 5%_—temperature of polymer degradation in air for 5% of weight loss; T_dec max_—degradation temperature at the highest rate in air atmosphere.

**Table 3:** Mechanical parameters of PBF and PBT based copolymers.

Sample	ρ [g/cm^3^]	Rm [MPa]	ε_b_ [%]	E [MPa]	σ_y_ [MPa]	ε_y_ [%]	H [ShD]
PBF-b-PTMG 65	1.24 ± 0.01	45.4 ± 0.9	477 ± 46	301 ± 61	14.4 ± 0.7	20.7 ± 0.5	52.2 ± 2.0
PBF-b-PTMG 65 A	-	48.1 ± 1.8	556 ± 83	192 ± 19	17.5 ± 0.4		-
PBF-b-PTMG 50	1.20 ± 0.01	47.8 ± 1.3	643 ± 84	113 ± 41	10.2 ± 0.1	26.0 ± 0.4	46.1 ± 1.4
PBF-b-PTMG 50 A	-	42.1 ± 1.2	654 ± 24	97 ± 31	12.6 ± 0.6		-
PBF-b-PTMG 35	1.14 ± 0.01	38.1 ± 3.0	657 ± 34	78 ± 19	7.4 ± 0.2	40.6 ± 0.6	40.0 ± 1.1
PBF-b-PTMG 35 A	-	29.8 ± 3.0	606 ± 47	40 ± 9	-		-
PBT-b-PTMG 65	1.22 ± 0.01	34.5 ± 0.6	578 ± 48	306 ± 26	18.4 ± 0.04	27.3 ± 0.6	50.4 ± 0.6
PBT-b-PTMG 65 A	-	21.2 ± 1.1	138 ± 46	298 ± 24	22.2 ± 0.3		-
PBT-b-PTMG 50	1.17 ± 0.01	28.0 ± 1.0	763 ± 88	125 ± 12	11.0 ± 0.1	29.7 ± 0.7	45.3 ± 0.9
PBT-b-PTMG 50 A	-	23.3 ± 0.6	760 ± 75	103 ± 7	12.1 ± 0.2		-
PBT-b-PTMG 35	1.13 ± 0.01	25.5 ± 3.4	741 ± 70	95 ± 3	9.5 ± 0.3	30.0 ± 0.6	41.7 ± 0.6
PBT-b-PTMG 35 A	-	24.4 ± 1.7	688 ± 60	107 ± 6	-		-

The values determined for samples after one month of storage and after annealing. ρ—density at 23 °C; Rm—tensile strength; ε_b_—elongation at break, E—tensile modulus, σ_y_—yield stress, ε_y_—elongation at yield, H—Shore D hardness. The values are the average of at least six tests and the standard deviation.

## Data Availability

The data presented in this study are available on request from the corresponding author.

## Supplementary Materials

The following supporting information can be downloaded at: https://www.mdpi.com/article/10.3390/molecules28072962/s1, Figure S1: FTIR spectra of all investigated copolymers; Figure S2: HNMR spectra of PBT-b-PTMG copolymers; Figure S3: TGA thermograms of PBF and PBT copolymers received for samples subjected to thermo-oxidative atmosphere, Figure S4: WAXS diffractograms of PBF-b-PTMG injected samples received after a few weeks of storage with calculated crystallinity degrees. Figure S5: The representative uniaxial stress–strain paths of (a) PBF and (b) PBT copolymers tested in 48 h and after one week from processing, Figure S6: The cyclic stress–strain paths for PBT (a) and PBF (b) copolymers. Samples after one month of storage. Table S1: The chemical composition and molecular weights of PBT-b-PTMG copolymers; Table S2: Mechanical parameters of PBF and PBT copolymers in 48 h and one week after injection moulding.
