# Supplementary material for: Hytrel-like Copolymers Based on Furan Polyester: The Effect of Poly(Butylene Furanoate) Segment on Microstructure and Mechanical/Elastic Performance
Source: Molecules. 2023 Mar 26;28(7):2962. doi: 10.3390/molecules28072962 (PMC10095974; doi:10.3390/molecules28072962)
Supplement: Supplementary file 1 [file molecules-28-02962-s001.zip › molecules-2288336-supplementary.pdf]

Supporting Information

Hytrel–Like Copolymers Based on Furan Polyester: The Effect of Poly(Butylene Furanoate) Segment on Microstructure and Mechanical/Elastic Performance

Magdalena Kwiatkowska[1,*], Inez Kowalczyk[1], Zbigniew Rozwadowski [2], Elżbieta Piesowicz[1] and Anna Szymczyk[3,*]

1 Department of Materials Technology, West Pomeranian University of Technology in Szczecin, Piastow Av. 19, 70-310 Szczecin, Poland
2 Department of Inorganic and Analytical Chemistry, West Pomeranian University of Technology in Szczecin, Piastow Av. 42, 71-065 Szczecin, Poland
3 Department of Physics, West Pomeranian University of Technology in Szczecin, Piastow Av. 17, 70-310 Szczecin, Poland
* Correspondence: magdalena.kwiatkowska@zut.edu.pl (M.K.); anna.szymczyk@zut.edu.pl (A.S.); Tel.: +48-91-449-45-89 (M.K.); +48-91-449-41-73 (A.S.)

Figure S1

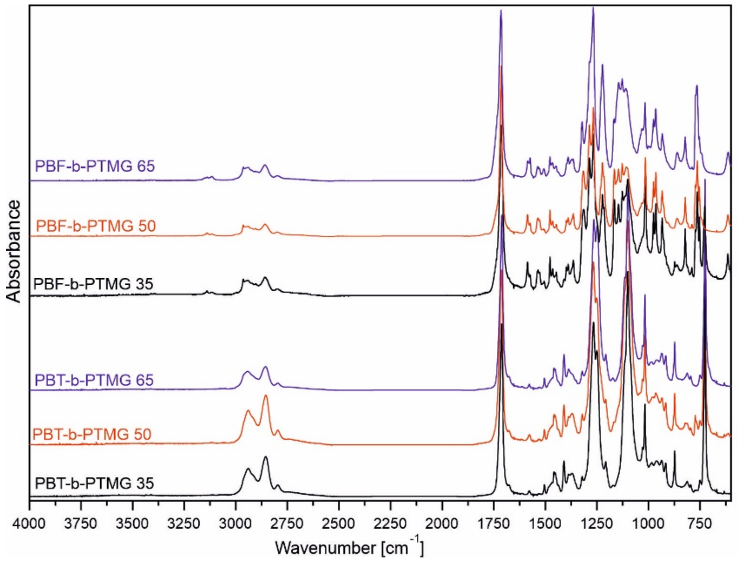

FIGURE S1. FTIR spectra of all investigated copolymers

Figure S2

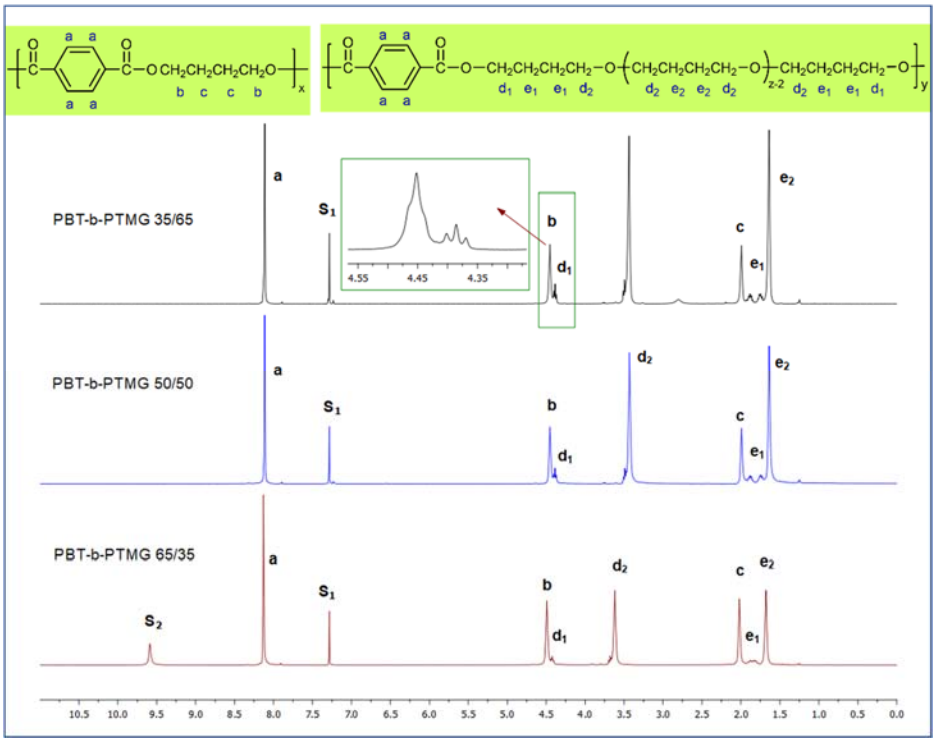

FIGURE S2. ^{1}H-NMR spectra of PBT-b-PTMG copolymers in $CDCl_3$ (s_1)+ drops of CF_3COOD (s_2).

The chemical composition of PBT-b-PTMG copolyesters was confirmed by ^{1}H-NMR spectroscopy (Figure 2S). The peaks at position of 3.62 ppm (peak ***d***$_2$, -OC**H**$_2$-CH$_2$-CH$_2$-C**H**$_2$O-) and 1.64 ppm (peak ***e***$_2$, -OCH$_2$-**CH**$_2$-**CH**$_2$-CH$_2$O) are assigned to the protons from PTMG flexible blocks, while the peaks at 8.11 ppm (peak ***a***, aromatic protons), 4.45 ppm (peak ***b***, -COO-C**H**$_2$-CH$_2$-CH$_2$-C**H**$_2$-OOC-) and 1.99 ppm (peak **c**, -COO-CH$_2$-C**H**$_2$-C**H**$_2$-CH$_2$-OOC-) to the protons from PBT rigid segments, respectively. At 4.39 ppm (peak ***d***$_1$) and at 1.88 ppm (peak ***e***$_1$). The peaks at 4.39 ppm (***d***$_1$) is and at 1.88 ppm (***e***$_1$) corresponds to the flexible PTMG blocks ends connected with terephthalate unit of the PBT segment.

The real fractions of the PTMG segments (**Table S1)** in copolymers were calculated based on the characteristic peaks' integral intensities, according to Equation (S1):

$$W_{PTMG}(wt\%) = \frac{\left(\frac{I_{e1}}{4} x\ 88\right)+\left(\frac{I_{e2}}{4}\ x\ 72\right)}{\left(\frac{I_a}{4}\ x\ 220\right)+\left(\frac{I_{e2}}{4}\ x\ 72\right)} 100\% \qquad (S1)$$

where: I_a, I_{e1}, and I_{e2} are the integral intensities of a, e_1 and e_2 resonances on ^{1}H-NMR spectra, whilst 220, 72, and 88 are the molecular weights of repeating units in PBT and PTMG blocks, respectively.

Table S1. The chemical composition and molecular weights of PBT- *b*-PTMG copolymers.

Sample	In feed			^{1}H-NMR								
	DPx mol	**W_{PBT} wt %**	**W_{PTMG} wt %**	I_a	I_b	I_c	I_{d1}	I_{d2}	I_{e1}	I_{e2}	**W_{PBT} wt %**	**W_{PTMG} wt %**
PBT-b-PTMG 65	9.54	65	35	1.00	1.00	0.97	0.11	1.21	0.26	1.12	**65.56**	**34.44**
PBT-b-PTMG 50	5.14	50	50	1.00	0.92	0.90	0.21	2.38	0.23	2.57	**49.59**	**50.41**
PBT-b-PTMG 35	2.77	35	65	1.00	0.80	0.83	0.25	3.22	0.55	3.26	**38.10**	**61.90**

DPx – degree of polymerization of PBT block, W_{PTMG} – weight content of flexible block, W_{PBT} – weight content of rigid blocks

Figure S3

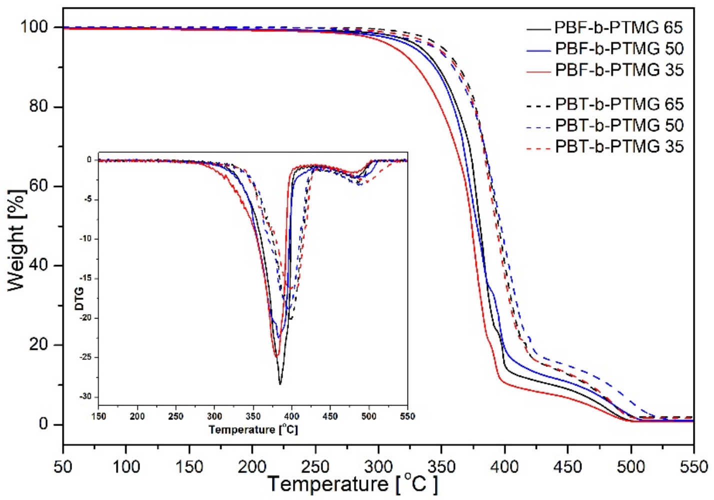

FIGURE S3. TGA thermograms of PBF and PBT copolymers received for samples subjected to thermo – oxidative atmosphere

Figure S4

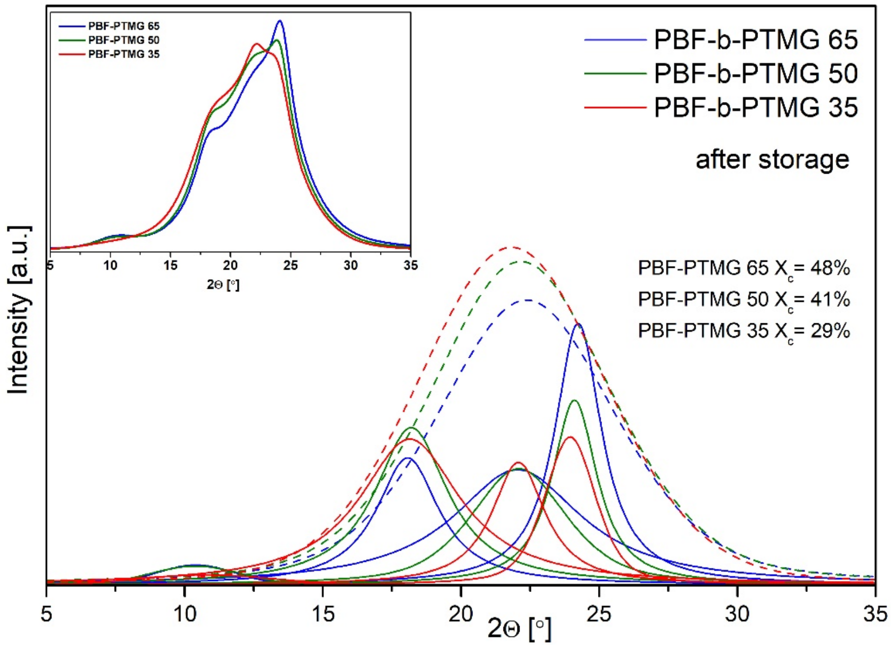

FIGURE S4. WAXS diffractograms of PBF-b-PTMG injected samples received after four weeks of storage with calculated crystallinity degrees.

Figure S5

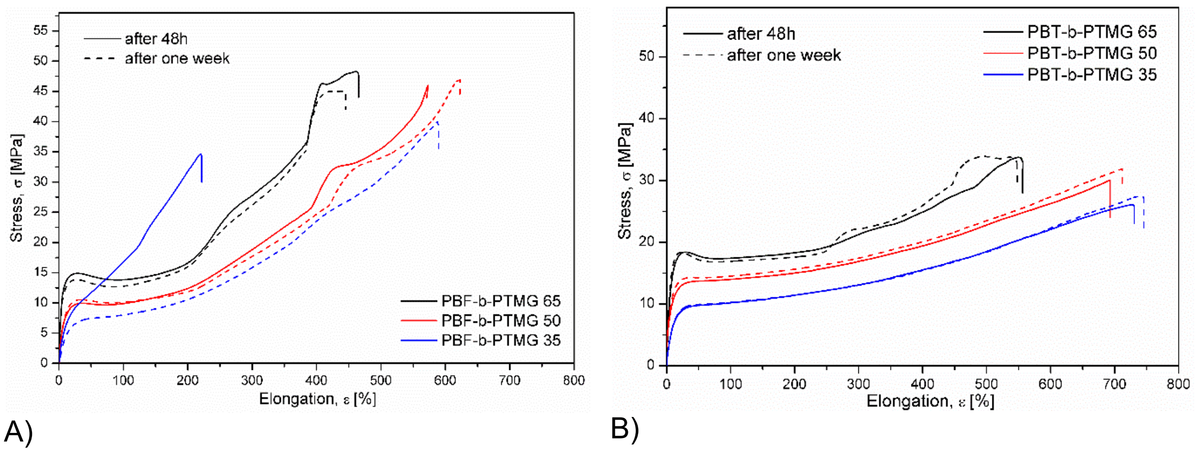

A) B)

FIGURE S5. The representative uniaxial stress – strain plots of A) PBF and B) PBT copolymers tested in 48h and after one week from processing

Table S2. Mechanical parameters of PBF and PBT copolymers in 48h and one week after injection molding

	After 48h				After one week			
**Sample**	**Rm [MPa]**	**ε_b [%]**	**E [MPa]**	**σ_y [MPa]**	**Rm [MPa]**	**ε_b [%]**	**E [MPa]**	**σ_y [MPa]**
PBF-b-PTMG 65	48.1 ± 0.9	487 ± 46	217 ± 58	14.8 ± 0.1	44.7 ± 1.1	468 ± 48	143 ± 33	14.2 ± 0.4
PBF-b-PTMG 50	45.1 ± 5.0	576 ± 18	111 ± 25	9.9 ± 0.1	47.8 ± 3.5	645 ± 50	79 ± 12	10.5 ± 0.2
PBF-b-PTMG 35	34.7 ± 2.0	230 ± 24	103 ± 24	9.6 ± 0.3	41.1 ± 1.0	597 ± 59	86 ± 24	7.5 ± 0.1
PBT-b-PTMG 65	33.8 ± 0.3	556 ± 36	296 ± 64	18.3 ± 0.1	34.4 ± 0.9	560 ± 38	287 ± 50	18.6 ± 0.1
PBT-b-PTMG 50	31.3 ± 0.6	692 ± 34	171 ± 29	13.7 ± 0.1	32.3 ± 0.6	712 ± 33	135 ± 10	14.2 ± 0.1
PBT-b-PTMG 35	25.1 ± 2.7	669 ± 69	133 ± 25	9.7 ± 0.1	25.4 ± 2.6	713 ± 84	79 ± 6	9.3 ± 0.4

Figure S6

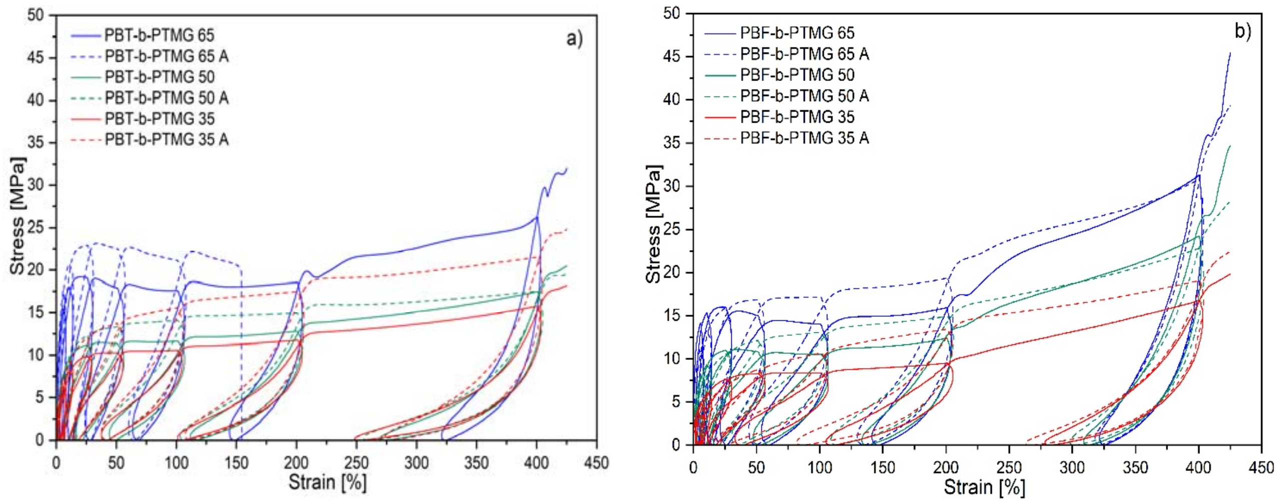

FIGURE S6. The cyclic stress – strain paths for PBT (a) and PBF (b) copolymers. Samples after one month of storage. Dash lines refer to annealed samples.
